# Embedded Target Filler and Natural Fibres as Interface Agents in Controlling the Stretchability of New Starch and PVOH-Based Materials for Rethinked Sustainable Packaging

**DOI:** 10.3390/ma15041377

**Published:** 2022-02-13

**Authors:** Doina Dimonie, Bogdan Trica, Celina Damian, Roxana Trusca

**Affiliations:** 1 National Institute for Research & Development in Chemistry and Petrochemistry—ICECHIM, Spl. Independentei 202, 060021 Bucharest, Romania; ddimonie@yahoo.com; 2 Faculty of Applied Chemistry and Materials Science, Polytehnic University of Bucharest, Spl. Independentei 313, 060042 Bucharest, Romania; celina.damian@upb.ro; 3 Department of Science and Engineering of Oxide Materials and Nanomaterials, Polytehnic University of Bucharest, Spl. Independentei 313, 060042 Bucharest, Romania; truscaroxana@yahoo.com

**Keywords:** starch, structuring, rethinked packaging, miscibility, fillers, compatibilizer

## Abstract

A structuring solution converting starch into a multiphase polymeric material was obtained through a melt compounding sequence, which can be irreversibly shaped by thermoforming into rethinked, sustainable packaging, based on the physical modification of starch with polyvinyl alcohol (PVOH), target fillers, (CaCO_3_ and wood flour), and a good plasticizer compatible with the polar components. Polymeric material can be thermoformed if it can be stretched without breaking in the positive temperature range, have functional properties required by the application, and keep its shape and properties after stretching for more than six months. The properties of the selected quaternary starch-based compound, fulfil the requirements for a thermoformable polymeric material due to the chemical compatibility between the components and the compounding in a selected procedure and optimal conditions wich ensure a comfortable miscibility. Most likely, the obtained miscibility can be explained only by the arrangement of the wood flour at the interface, where it acts as compatibilizer with a main role in structuring the new starch-based materials. The compatibilizer role of the wood flour was proved for the quaternary selected blend by the changing of the thermal degradation mechanism, from one with two stages for binary and tertiary blends, to one consisting of a single stage: decreasing till elimination of morphological defects, the reproducibility of the mechanical properties, stretching without breaking, and dimensional stability after stretching. Future studies will aim to achieve rethinked packaging for applications that require higher strength properties.

## 1. Introduction

In a changing world, plastic production is projected to increase four-fold in tonnage by 2050. As a result, plastic packaging waste alone is expected to double within the next fifteen years and quadruple by 2050 [1,2,3,4]. For complex reasons, currently almost 72% of plastic packaging worldwide is not recovered [4]. To eradicate plastic waste and pollution at the source, and to decouple plastics from fossil feedstocks by adopting renewable sources, a new plastic circular economy and bioeconomy concept can be formulated with the following main goals: less plastic (rethinked plastic applications), better plastic (better correlation of the functional properties with the application requirements, increased recyclability for pre-and post-consumer plastic goods) and no plastic use, and where possible, the use of alternative materials close to nature, or even from nature (e.g., natural fibres) [5]. Rethinking plastics will be achieved by improving the circularity of plastic packaging by means of using materials not based on compounds of concern and encouraging the use of compostable packaging, while conferring sustainability to plastic packaging by making them reusable, recyclable or recoverable by considering mainly renewable resources [6,7].

Starch-based materials are considered to be the future of sustainable and rethinked packaging [8]. Starch is a cheap polymer originating from annually renewable resources and consists of two polymers, amylose and amylopectin. Both are based on a large number of repeated glucose units joined by glycosidic bonds in linear macromolecules (amylose), or other by cluster type (amylopectin) [9,10]. After overcoming the existing drawbacks, such as water resistance, high brittleness, poor processability, etc., starch might be used for designing environmentally friendly polymeric materials for various applications [11,12,13]. Attempts to attenuate these drawbacks mainly consider removing the hydrogen bonds between the hydroxyl groups from its macromolecules via plasticizing [14] and/or physical or chemical modifications by adding biodegradable synthetic or renewable polymers [9,15,16,17,18,19], including poly (vinyl alcohol) (PVOH) and the establishment of other hydrogen bonds, this time between its macromolecules and those of the used polymeric modifiers. [9,15,16,17].Starch-PVOH blends are one of the most popular biodegradable multiphase polymeric materials, known as compounds, composites and nanocomposites [20,21,22,23,24,25,26]. PVOH is a non-renewable, biodegradable petroleum polymer with controlled water solubility, excellent mechanical properties and good thermal stability and processability [22,23,24,25]. Composites, starch/PVOH/Na + MMT, have improved tensile strength and better elongation properties [26].

Fillers are inorganic additives, less frequently organic, used in plastic formulation for controlling [27,28,29,30,31,32,33,34,35] certain physical and mechanical properties while reducing the price [27]. Calcium carbonate (CaCO_3_) is the most inexpensive, inorganic natural mineral filler [29,30,31,32,33,34,35] utilized for plastics [29,32,33,34] and many other materials [30,31]. Natural fibres are useful in polymer formulation for lowering weight, improving impact and flexural properties, sound insulation, moldability, surface appearance and conferring a texture similar to solid wood, and increasing durability, while remaining environmentally friendly, etc. [35].Wood fibres are natural composites with several layers of cellulose fibrils, embedded by lignin and hemicellulose in a complex physical structure containing 40–50% cellulose, 20–30% hemicellulose, 25–35% lignin, max. 25% extractives [36]. Cellulose is a linear polymer with repeated units of anhydro-D-glucopyranose connected by β−1,4-glycosidic bonds [37]. Hemicelluloses belongs to the heterogeneous polysaccharides family with complex molecular structures [38], and lignin is an alkyl-aromatic polymer with many lateral chains and various polar functional groups [39].

The shaping process of the polymeric materials by thermoforming into goods generates notable morphological changes into the plate’s structure as a function of material characteristics, the temperature at which the plate was heated in view of thermoforming and the applied force [40]. The obtained thermoformed goods are designed for various industrial and non-industrial applications, including packaging (e.g., food industry as food storage containers, etc.). Concerning the subject of designing and achieving the mentioned objective through melt compounding methods, polymeric materials with good thermoformability are not found in the literature, either for non-renewable source-based polymers or for renewable ones.

The purpose of this work was to design and identify a structuring solution, using the compound technique, to convert starch into a multiphase polymeric material, so that it can be irreversibly shaped by thermoforming for rethinked, sustainable packaging.

## 2. Materials and Methods

### 2.1. Designing of New Starch Based Compounds

To find a structuring solution for converting starch into a multiphase polymeric material, which can be irreversibly shaped by thermoforming into rethinked sustainable packaging, starch was physical modified with polyvinyl alcohol (PVOH), target inorganic (CaCO_3_) and organic (wood fibre) fillers and a good plasticizer compatible with the polar components, glycerol. PVOH was selected for its very good processability and capability to ensure the needed extensibility for feasible thermoformability. The two target fillers were preferred for their effect on controlling thermal behaviour and due to the convenient ratio between the elastic and plastic deformation [41], which ensured that the new material could be stretched without breaking. Furthermore, they improved dimensional stability, had a structuring effect [42,43], and reduced the price [44,45,46]. The wood fibre can be responsible for the surface appearance, moldability and specific weight [47], whilst glycerol improves processability, extensibility, miscibility and thermal stability. From these five components, which have similar chemical structures and a high content of polar groups, mainly of the hydroxyl type [10,34,36,37,38,39,42,44,45,46,48,49,50], three of them are of renewable grade and have good compostability [9,10,36,37,38,39,49,50]. Near its renewable origin, glycerol can be achieved by refining residues from biodiesel manufacturing [50].

To design and determine new thermoformable polymeric materials based on starch involves the identification of those compositions and the methods which ensure the proper behaviour of the new materials throughout the entire chain, from conception until their completion as thermoformable items. At the same time, an understanding of its behaviour over time, in addition the recycling of pre-and post-consumer wastes is also important to consider. This polymeric material must meet certain properties, both in the molten and solid state, which will be described below. In the molten state, the melt fluidity and the melt resistance must allow good homogenization without degrading the used polymers and the other components [34,48]. The targeted properties in the solid states mainly concern thermal behaviour to ensure that the thermoforming is carried out at positive temperatures without breaking, and the obtained object keeps its shape, even during hot summers, the dynamic-mechanical properties with which the optimal ratio between the elastic and the plastic deformation (as an expression of thermoformability), can be chosen. Additionally, in the solid state, is required the selection of those formulations with moderate extensibility as indicators of the material’s ability to be thermoformed without breaking and the ability of the new items to keep their shaped during usage and whit smooth appearance, conditions necessary both for the plates and the thermoformed items. Breakage of the plates during thermoforming can occur when the materials’ extensibility is too small, as a consequence of an improper ratio between elastic and viscous deformation—the viscous component being too high and/or a large undesirable residual stress (as a consequence of the improper plates achieving conditions). When the elastic deformation is too high, the thermoforming can no longer occur as the material is too ductile.

A key step to obtain the thermoformable starch-based polymeric materials was to establish the compounding ratios between the selected components, and to identify the optimal melt compounding sequence together with the working conditions which ensure the targeted properties and their level. The characterization methods which highlight the studied properties of the new starch based multiphase polymeric materials were selected.

### 2.2. Experiments

In order to achieve thermoformable starch-based materials, the following experimental procedure were performed: (a) new compounds were obtained through a classical Brabender (temperature between 130 °C and 150 °C, speed of 75 rpm, compounding time of 4 min and 20 min)—roller (temperature of 40 °C and 100 °C, speed1/speed2 of 1.2) method, (b) preliminary characterization (surface appearance, breaking strength), (c) preliminary compounds selection (following the breaking strength and the surface appearance), (d) extrusion compounding and granulating of the selected compounds, (e) pressing plates with different thickness (145 °C temperature, 10 min pressing; 10 min cooling, 100 kN), f) thermoforming (variation of the pressing pressure between 50 kN and 200 kN with the thermoforming time between 5 min. and 10 min), (g) time following the thermoformed items. The extrusion compounding was made using a Gotffert laboratory extruder (43 mm diameter and L/D of 37) by working in the following conditions: T = 120–150 °C, extrusion speed of 45 rot/min, 270 bar of pressure, 320 bar back pressure.

To improve the miscibility of the components, a procedure in two stages was tested: one for pre-gelling using the conveying screw and the next one for intensive melt homogenization with the compression screw (Table 1). The difference between the two screws is related to the height and distance between the screw coils in its three areas: feeding, compression and conveying (Table 1). In both extrusion procedures, all the other extrusion conditions, namely T = 110–130 °C, 270 bar pressure, 320 bar back pressure, were the same.

The improvement of the plate morphologies isotropy was attempted in a pressing experiment at four different pressures (50 kN, 100 kN, 150 kN and 200 kN) and identical to all the other conditions (130 °C temperature, 10 min preheating, 20 min pressing, 10 min cooling), which finally showed the optimal pressure to eliminate the results dispersion.

The thermoforming behaviour was tested according to Section 2.3.2.

In the experiment which was carried out, the following materials were used: corn starch powder from Sigma Aldrich (Darmstadt, Germany) with 70 °C glass transition, 167 °C melting temperature, 30% amylose−70% amylopectine, partially hydrolysed PVOH provided by Du Pont (Wilmington, DE, USA) with 88% hydrolysis degree, 12% residual polyvinyl acetate,1200 polymerization degree, 68 °C glass transition, glycerol from Brenntag (Essen, Germany), 290 °C boiling point, 160–170 °C flash points, natural micrometric CaCO_3_ from Nuwen (Dinard, France), with particle sizes ranging between 535.7 nm and 3.01 µm, compostable soft wood flour from LA. SO. LE. (Percoto, Italy), “150” grade with average particle diameter 150/200, average L/D = 4.8, vacuum dried, at 73 °C overnight).

Binary (PVOH-starch), ternary (PVOH-starch- CaCO_3_) and quaternary (PVOH-starch- CaCO_3_- soft short wood fibre) compounds were achieved in the following combinations of components: (10–40%) starch, (40–60%) PVOH, (2–15%) glycerol, (0–67%) CaCO_3_, and (12–30%) soft wood flour.

### 2.3. Characterization of the New Starch Based Multiphase Materials

#### 2.3.1. Thermal, Mechanical, Dynamic-Mechanical, Physical Properties

a Thermal behaviour:

- Thermogravimetric analysis (TGA) was operated on TGA Q500 system (TA Instruments) under nitrogen atmosphere. The samples were heated from room temperature to 600 °C at a heating rate of 10 °C/min and a nitrogen gas flow rate of 90 mL/min in the furnace. The derivative of TGA curves was obtained using TA analysis software.
- Differential Scanning Calorimetry (DSC) curves were recorded on a Netzsch DSC 204 F1 Phoenix equipment. The samples were subjected to analysis from 0 to 200 °C using a heating rate of 10 °C/min, then cooled to 0 °C with a cooling rate of 10 °C/min and finally heated to 300 °C using a rate of 5°/min under nitrogen atmosphere (20 mL/min flow rate).
- Dimensional stability—exposure in an oven with air circulation, for 10 min, at temperatures between 80–120 °C of specimens with width of 20 mm and a length of 50 mm and the calculation of the percentage variation of the width after the expiration of the test period by reporting the difference between the width after temperature keeping and the initial one at the initial value.

b Dynamic-mechanical and mechanical behaviour:

- Dynamic mechanical analysis (DMA) was carried out on a TRITEC 2000 B (Triton Technologies) in a single cantilever mode from 60 to 100 °C with a frequency of 1 Hz and a heating rate of 5 °C/min. During testing, the dynamic mechanical property parameters of storage modulus, loss modulus and loss factor were recorded from negative to positive temperatures, in ranges of interest for thermoforming. Because the range of very low negative temperatures is not of interest for thermoformed packages, the dynamic-mechanical analysis of the PVOH-starch compounds with CaCO_3_ and of those with CaCO_3_ and wood flour was made starting from −25 °C to 160 °C. The DMA curves describe the elastic (storage modulus) and plastic deformations (loss modulus) of the studied material and also their glass transition represented by the temperature of intersection of the curves representing the temperature variation of loss modulus and tan (Δ).
- Mechanical properties—ASTM D 638 considering mainly the stress—extensibility registration as expression of the materials thermoformability [51].

c Physical properties:

- Density—ISO 1183 by using an analytical balance [52];
- Hardness—ASTM D2240 by using a durometer Shore A/D device to measure the material’s resistance to indentation [53].

d Rheological properties of melts

The melt rheological properties were measured according to [29,30,31,32,33,34,35,54,55], on a DYNISCO 4000 LMI indexer which displayed the following properties: melt flow index, dynamic viscosity, shear rate. The indexer was equipped with a nozzle having a 2.09 ratio between height and diameter (h/D) The measurements were carried out at four temperatures (from the range of 125–170 °C) and four loads (between 2.16 kg and 10 kg) to ensure the minimum number of experimental points which can validate the conclusions. It was considered that the flow of the melt was stationary if the extrudates surface had a smooth appearance and the shear rate-temperature dependence was concave shaped.

#### 2.3.2. Thermoforming Behaviour

The plates at two values of thickness were heated at different temperatures (80 °C and 100 °C) for 5–10 min, in an air-circulating oven, stretching at different pressures for different times, cooling for 30 min by using two positive moulds, one with a diameter of 31.5 mm and another of 60 mm. The stretching pressure was varied between 20 kN and 250 kN. The stretching ratio (R_str_) was calculated as the ratio between the final wall thickness of the thermoformed plate and the initial thickness of the plate that was subjected to thermoforming. The ratio 1/R_str_, which highlights the average reduction in the wall thickness of the thermoformed items, was also calculated.

#### 2.3.3. Time Behaviour

Thermoformed objects were stored for more than 1 year, at room temperature, on storage shelves. They were periodically inspected for shape changes, cracks appearance and breaking behaviour which was manually tested.

## 3. Results

### 3.1. Thermal Behaviour

The Starch-PVOH binary blends plasticized with 27–42% glycerol show multiple small transitions starting from −87 to 95 °C, two at negative temperatures, one in a wider range of 10–20 °C and one, at positive temperatures in a narrower range of 39–61 °C. Depending on the plasticizer concentration the melting occurs between 73–94 °C (Table 2 and Table 3). The very low melting enthalpy of 0.2–0.4 J/g reveals a small crystallinity, which means that these compounds mainly have an amorphous morphology. The phase transitions are related to the changes in molecular mobility, in addition to the ordering–disordering processes, which can take place most likely as a consequence of the unstable (metastable) equilibrium characterizing these compounds [40]. By the proper selection of the melt compounding conditions, miscibility of these compounds can be really improved, as it was demonstrated in literature [34,55].

**Figure 1:**
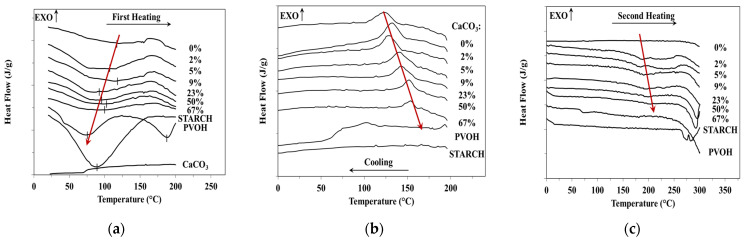
DSC thermograms of ternary starch-based blends with variable CaCO_3_ content: (**a**)-first heating; (**b**)cooling between first and second heating; (**c**)second heating.

The thermal behaviour of the PVOH-starch-CaCO_3_ ternary blends with variable filler content is revealed by Figure 1, with results also shown in Table 3. The increase in CaCO_3_ content diminishes the evaporation heat from 170 J/g to 91.3 J/g and of the evaporation temperature maximum at 122.3 °C (Figure 1a) up to 92.60 °C (Table 3). Due to the variable CaCO_3_ content, the melting is characterized by small peaks and a wide range (Figure 1b) with the maximum melting temperature increased from 180 to 202.7 °C (Figure 1b) with the melting enthalpy decreased from 104.4 J/g down to 16 J/g (Table 3). For the same reason, the crystallization temperature increases from 120.3 °C up to 153.5 °C (Figure 1c), while the crystallization enthalpy decreases from 75.6 J/g as far as 15.3J/g (Table 3). Consequently, increasing the CaCO_3_ concentration makes these compounds almost amorphous and slightly hygroscopic. If starch has a glass transition of 70 °C, PVOH of 68 °C and a strongly plasticized binary blend of −60 °C, due to the presence of CaCO_3_, the ternary blends consisting in all of these three components have this transition at temperatures above 100 °C, even up to 169.4 °C at 67% CaCO_3_ (Table 4).

**Figure 2:**
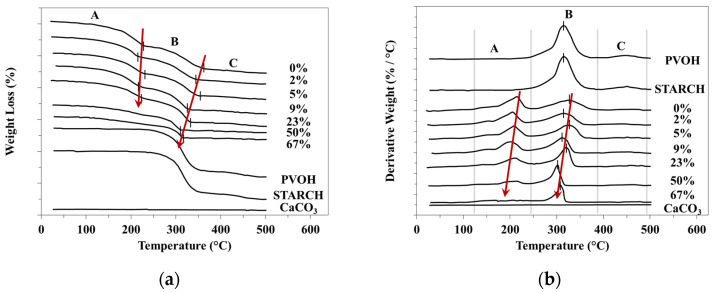
Degradation behaviour of ternary starch-based blends with different CaCO_3_ amount: (**a**)-TGA curves; (**b**) DTA curves.

Degradability of the PVOH-starch ternary compounds depends also on the CaCO_3_ concentration (Figure 2, Table 5). If the two base polymers, PVOH and starch degrade in a single step process, all the compounds with CaCO_3_ degrade in two steps, each stage with a maximum value at a slightly descending temperature if the CaCO_3_ quantity increases. The total weight-loss at 500 °C is of 5.8% for the compound without CaCO_3_ and of 39.3% for those with 67% filler (Table 5). These results show that the filler acts as a pro-degrading agent and, therefore, to avoid the degradation at melt compounding, proper stabilization must be achieved.

The presence of the 33% wood flour in the ternary blend of starch with 33% PVOH and 37% CaCO_3_ changes entirely the thermal behaviour: the wood flour decreases the crystallinity, as evidence of the melting and crystallization enthalpies, the reduction of the melting enthalpy being close to 87% compared to the blend without wood flour (Table 6).

**Figure 3:**
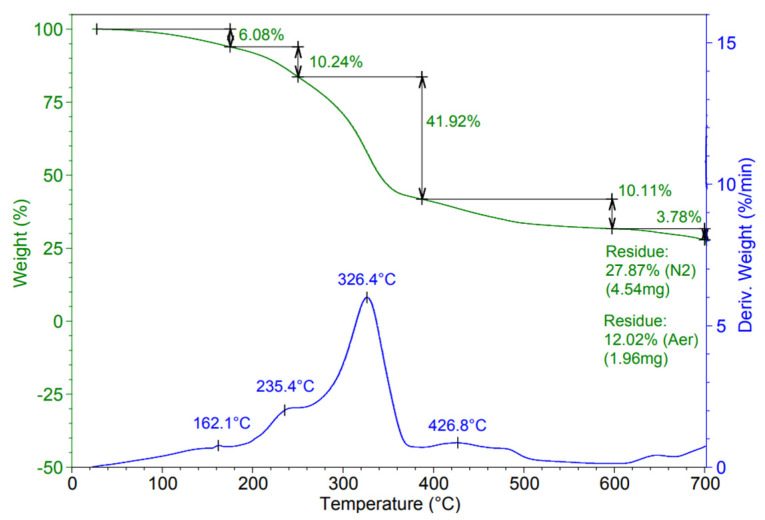
The thermal degradation of the selected quaternary starch-based compound.

Unlike the tertiary compounds which degrade following a two-stage mechanism (Figure 2), the quaternary containing supplementary wood flour (WF) degrades after a single-stage process with a maximum temperature of 326.4 °C and two small shoulders, one at 162.1 °Cand another at 235.4 °C (Figure 3). This blend loses 5% from its weight at 110 °C, namely about 20 °C lower than in the case of the compound without wood flour. The loss is 50% at 307 °C compared to 340 °C in the case of the compounds without wood flour, which means that this renewable filler accentuates the compound thermal degradation.

### 3.2. Dynamic-Mechanical Behaviour

**Figure 4:**
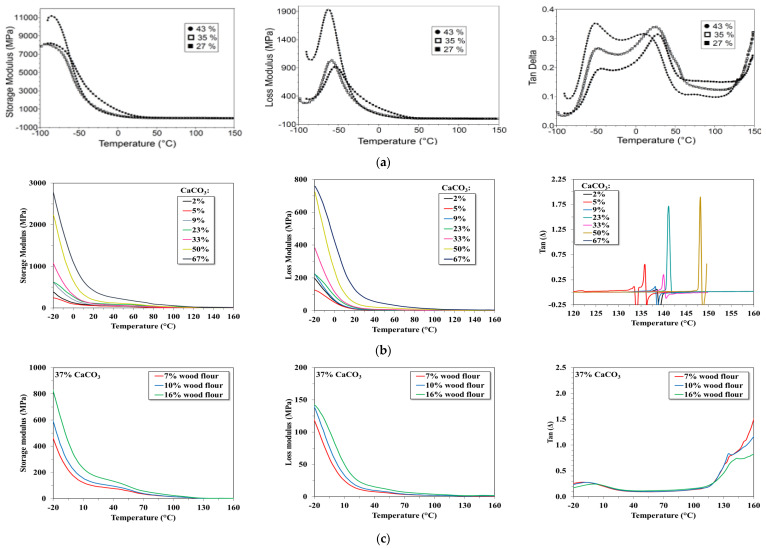
Dynamic-mechanical behaviour of binary, ternary, and quaternary starch-based blends (**a**) PVOH-starch; (**b**) PVOH-starch-CaCO_3_; (**c**) PVOH-starch-CaCO_3_- wood flour.

The DMA curves (Figure 4) confirm the conclusions which follow from the DSC registrations (Table 1 and Table 2) according to which the plasticized binary PVOH–starch blends have transitions in the negative and positive temperature range, a main one around 50 °C and another at about 50 °C (Figure 4a). These transitions are more intense for the compound with 43% plasticizer. According to the shape of the DMA curves, in this temperature range, the ternary (Figure 4b) and also the quaternary composites (Figure 4c) contain more amorphous polymers. The values of the glass transitions of the ternary blends indicated by the DMA curves (Figure 4b) are comparable with those obtained from the DSC thermograms (Table 3). The glass transition of the quaternary and tertiary compounds is almost similar, which means that they are in the solid state on the positive range of temperature: those with CaCO_3_ up to 140 °C and until 120 °C, the ones with both target fillers (Figure 4b, c). These results have proven that due to the fillers, the thermal behaviour of the resulting tertiary and quaternary blends of interest for thermoforming has been achieved.

At the same temperature of about 20 °C, the elastic deformation (storage modulus) is greatly diminished by the presence of CaCO_3_, from about 8000–11,000 MPa for binary blends, at 500–2800 MPa for ternary blends, more or less depending on the filler’s concentration (Figure 4a,b). However, if beginning with around 0 °C, the binary compounds no longer elastically deform (the storage modulus has very small values), the CaCO_3_ presence makes possible the elastic deformation of the ternary compounds at positive temperatures, up to 100 °C −120 °C, (Figure 4b). The influence of CaCO_3_ on the PVOH-starch compounds is spectacular, not because of the magnitude of the elastic deformation but in making it possible at interesting temperatures for thermoforming, namely in the positive range of temperatures. The high damping factor (tan Δ) between 40 °C up to 120 °C, demonstrates the ability of the new materials to absorb, in the solid state, mechanical energy without plastic deformation. In other words, it seems that if the PVOH–starch blends cannot be thermoformed, the same blends with CaCO_3_, could be turned into finished goods by such a procedure, as indicated also by the storage and loss moduli and the high values of the damping factor (tan Δ). Considering the variation of the loss modulus with the filer’s concentration, it seems also that the blends with CaCO_3_ show much higher viscous deformations (Figure 4b) than the blends without filler (Figure 4a). However, it must not be forgotten that the loss modulus is also controlled by the insufficient distribution of the filler into the polymeric matrix.

### 3.3. Mechanical Properties

**Figure 5:**
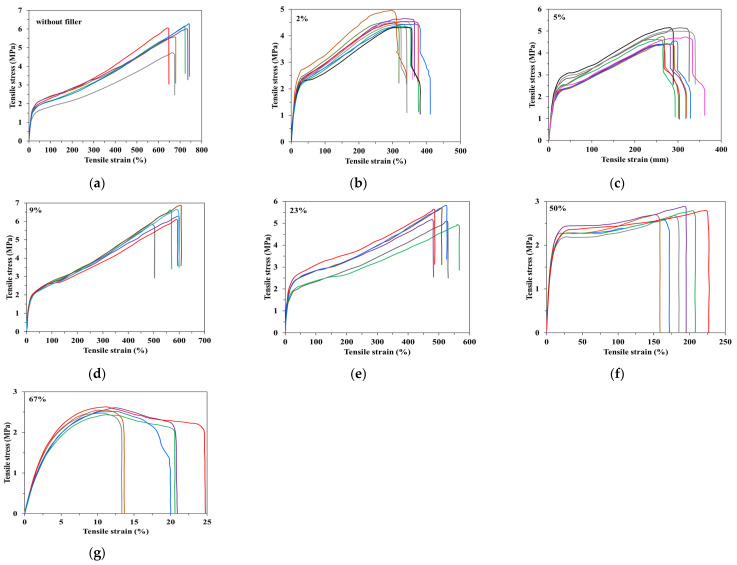
Influence of the CaCO_3_ concentration on the stress–strain dependence of binary and ternary starch-based blends (**a**)without filler; (**b**) 2%; (**c**) 5%; (**d**) 9%; (**e**) 23%; (**f**) 50%; (**g**) 67%.

In addition to their functional importance, the stress–strain rheograms (Figure 5) are a good illustration of the filler’s effect on the thermoplastic materials behaviour [56,57,58]. The experimental results show that the binary blends have mechanical behaviour between rigid and flexible polymeric materials with a tensile breaking strength between 6 and 7 MPa and tensile strain of 550–750% (Figure 5a). These blends present both yield strength (elastic limit), as for flexible materials [56,58] (close to 2 MPa),and continuous increasing of the tensile strain at achieving higher stress, as well as for the rigid ones [56,58]. At CaCO_3_ concentration between 2% and 23%, the mechanical behaviour keeps the same mixed character behaviour between rigid and flexible types with the following small changes: the slight increase in the yield strength, up to 2–3 MPa, the decrease in the tensile breaking resistance near 5–7 MPa and of the tensile strain up to values between 300% and 550% (Figure 5b–e). At 50% filler, the plateau area, when the deformation increases continuously, even the applied stress is constant, meaning that this compound behaves similarly to the flexible polymeric materials with high ductility [56,58] (Figure 5f). In this case, the yield strength remains around 2.5 MPa, the tensile breaking strength decreases to approx. 3 MPa and the tensile strain is ranged between 160% and 225%. The increase to 67% of the filler amount results in material change, but also mixed mechanical behaviour, more towards rigid materials, without yield strength but with a relative small plateau area, when at constant mechanical stresses of approx. 2.5 MPa, tensile strain reaches 13–25% (moderate ductility) (Figure 5g). In all cases, a relative dispersion of the results was observed, which requires a method to improve the filler’s dispersion into the base polymeric matrix.

**Figure 6:**
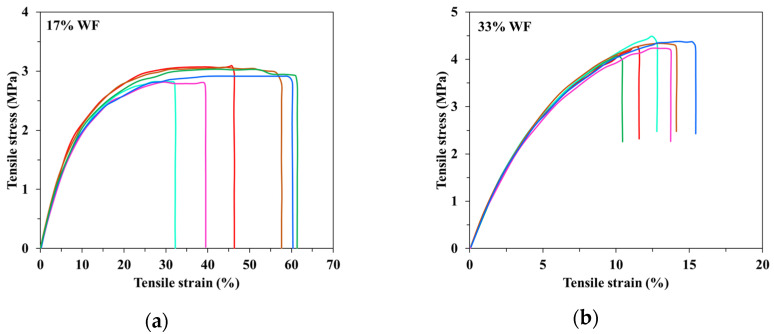
Influence of the wood flour concentration on the stress–strain behaviour of the quaternary starch-based blends (**a**) 15%; (**b**) 30%.

The addition of wood flour in the ternary compounds attenuates the rigid component of the mechanical behaviour found in ternary composites by slightly increasing the moderate ductile behaviour. The drawback of the poor distribution of the fillers into the polymeric matrix of these compounds demonstrated by the resulting dispersion, is kept too (Figure 6).

### 3.4. Melt Rheology Properties

**Figure 7:**
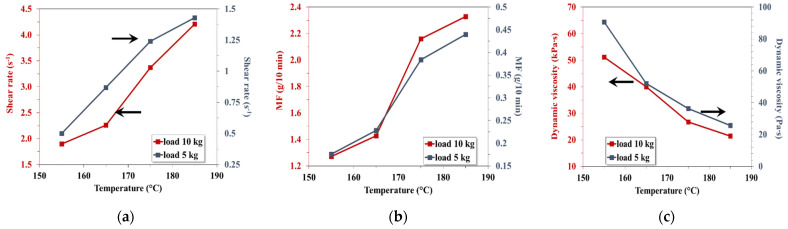
Rheological properties in the melted state of the selected quaternary starch-based blend (**a**) shear rate; (**b**) melt fluidity; (**c**) melt flow resistance.

In a previous article [34], the authors had shown that the same binary starch-based blends, obtained with the same materials in the same conditions develop, during melt flow at indexer, at temperatures between 145 °C and 175 °C and mechanically stress generated by 2.19–10 kg indexer’s loads, shear rates between 10 s^−1^ and 550 s^−1^. In the same conditions the fluidity is ranged from 3 g/10 in. to 130 g/10 min and the flow melt resistance between 200 Pa∙s and 7500 Pa∙s (Figure 7).

The new results prove that in the case of ternary starch-based compounds with 37% CaCO_3_, under the same flow conditions, the shear rate has values between 0.86 s^−1^ and 55.14 s^−1^, the fluidity has values between 0.44 g/10 min and 37.85 g/10 min and the dynamic viscosity between 1638 Pa∙s and 22,809 Pa∙s (Table 7).

**Figure 8:**
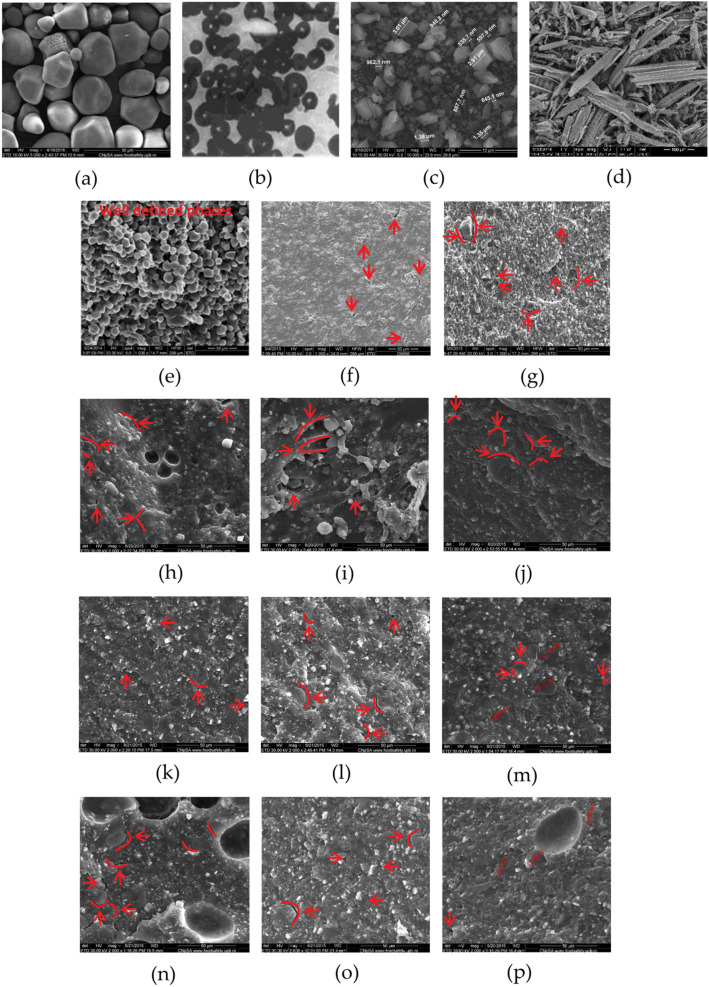

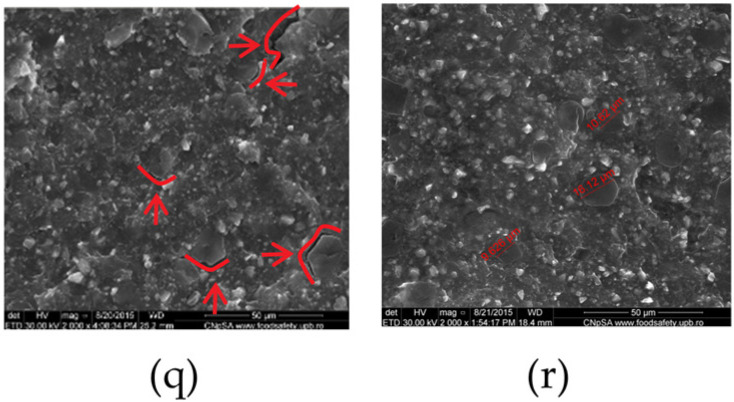
Targeted fillers: (**a**) starch; (**b**) PVOH; (**c**) CaCO_3_; (**d**) wood flour and SEM morphologies (1000×) of the binary and ternary compounds achieved after improving the miscibility through two procedures ((**e**)—binary blend; (**f**,**g**)—ternary blend/(**f**)—SEM surface/(**g**)—SEM fracture; (**r**)—quaternary blend. PROCEDURE 1—first stage: the conveying screw speed, rpm: (**h**)—5; (**i**)—20; (**j**)—50; second stage: the compression screw speed, rpm: (**k**)—40; (**l**)—40; (**m**)—40; PROCEDURE 2—first stage: the conveying screw speed: (**n**)—50 rpm;second stage: the compression screw speed, rpm: (**o**)—20; (**p**)—40; (**q**)—70) (the red arrows indicate the morphological defects).

The melt rheological properties of the quaternary selected starch-based blend, which has another component, wood flour, are fundamentally changed (Figure 8). In the same melt flow conditions, the fluidity increases from 1.2 g/10 min up to 2.4 g/10 min at 10 kg load and from 0.15 g/10 min to 0.45 g/10 min at 5 kg (Figure 8a). In the same temperature range, the dynamic viscosity rises from 20,000 Pa∙s as far as 60,000 Pa∙s at 10 kg load and from 15,000–80,000 Pa∙s at 10 kg load.

By comparative analysis of the above presented results, it can be ascertained that there is an almost ten-times decrease in the shear rate for the three categories of the studied starch-based blends in the following order: binary, ternary and quaternary. The shear rate represents the ratio between the speeds of the melt flows through a capillary divided by the gap size through which the flow occurs [48]. The melt will flow with a different rate depending both on the blend’s composition and the extrusion conditions. This means that the presence of fillers significantly affects the melt flow properties. The higher the fillers amount, the lower the flow rate will be and therefore a more intense homogenization effort is necessary to ensure a proper effect. This may be the explanation of the noticed, relatively large dispersion of the mechanical properties (Section 3.3) and, therefore, for the selected compounds, the optimal embedding conditions were identified. Melt fluidity, described by the MFI values, is another property that greatly differentiates the flow of the three compound categories. Due to the two fillers, the melt fluidity of the quaternary blend can be 50-times lower than in case of the binary blends. At the same time, the melt resistance to flow of the studied blends depicted by the dynamic viscosity values increases even 10 to 100 times, in the same order. These results show that the blends with CaCO_3_ or those with CaCO_3_ and wood flour must be achieved on a device or using a compounding sequence with a highly intensive melt homogenization effect.

### 3.5. Improving the Fillers Dispersion and Increasing the Packing Density of the Plates

As it was described in Section 2.1, to improve the component miscibility, all of those with isometric granulometry (Figure 8a–d), the binary and tertiary compounds were achieved by going through two different extrusion procedures. The SEM morphology (Figure 8a–d) reveals that the binary blend has a granular structure, consisting in the amylopectin residues, which cannot be spread into the matrix formed by the linear macromolecules of amylose and PVOH due to its branched character (Figure 8e). As the SEM micrographs prove, the granular character of the morphology of the ternary compound completely disappears. However, if this compound was achieved in a single stage extrusion procedure, then it has many voids on the surface and in fractures (Figure 8f, g). The fewest morphological defects such as cracks and fractures (marked with red arrows in Figure 8) have been formed at melt compounding in a two-stage procedure (procedure 1), by working with the conveying screw at low or medium speeds (5 rpm or 20 rpm) in the first stage and with the compression screw at medium speeds (40 rpm.) for the second one (Figure 8h,i,k,r). This procedure favours both the pre-gelling process as well as the melt homogenization.

**Figure 9:**
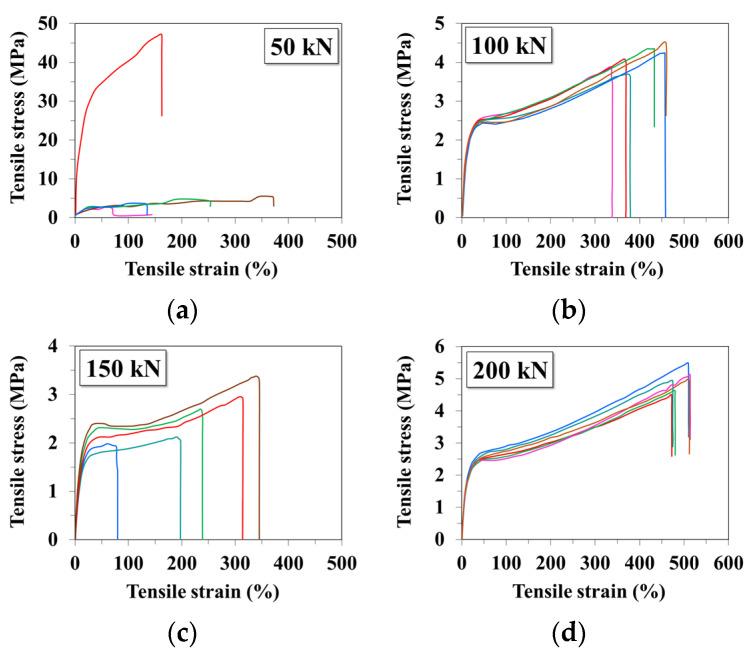
Dependence of the mechanical properties dispersion of the plates achieved from the selected ternary blends by the pressing pressure ((**a**)—50 kN, (**b**)—100 kN, (**c**)—150 kN, (**d**)—200 kN).

The quality of the plates that will be thermoformed can be very much improved if the packing density of the selected compounds is increased by pressing at a maximum pressure of 200 kN. In this way, it also eliminates the variation of the mechanical property’s values observed at pressing, namely at pressures lower than 200 kN (Figure 9).

### 3.6. Other Properties, Dimensional Stability

If the binary blend (PVOH with 30% starch) has a density of 1.231 g/cm^3^ and a hardness of 45°ShA, the density of the studied ternary blends is ranged between 1.23 g/cm^3^ to 1.72 g/cm^3^, and the hardness from 40°ShA to 70°ShA. The quaternary blend density is of 1.56 g/cm^3^. Dimensional stability refers to the ability of an item achieved from a certain polymeric material to maintain its size, even under various environmental conditions. It was found that at 80 °C the width of the sample achieved from the selected quaternary materials decreases by 0.003% and at 120 °C with 0.02%. This result can be a guarantee that the thermoformed items made from this selected compound do not change, over time, their shape, even in the hot summer.

### 3.7. Thermoforming Behaviour

**Figure 10:**
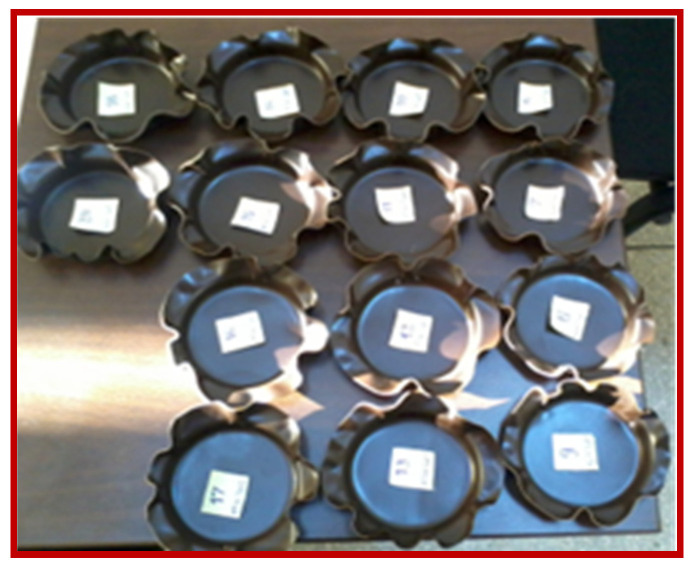
Items moulded from the selected starch-based material with target mineral and renewable fillers, thermoformed at 80 °C.

The optimal thermoforming parameters that lead to unbroken, complete shapes with smooth surfaces, appropriate appearance and meet all the required quality conditions were identified. These conditions have been met at values of 1.76–2.14 for the drawing/stretching ratio, for the positive moulder with 31.5 mm diameter and 1.67–2 for those with 60 mm diameter. Thinning the thickness of the plate by thermoforming was of 35–43% from the initial thickness. The optimum thickness of the needed plates for thermoforming was found to be 0.6 cm. A good reproducibility of the thermoforming behaviour of the selected quaternary compound was proved (Figure 10).

### 3.8. Time Behaviour

The observation of the time behaviour showed that, during a year, the thermoformed items do not change their shape, do not crack and do not break. These results can be explained only as a consequence of a very good miscibility between the components of the selected compounds, which was reached.

## 4. Discussions

The selected structuring solution for converting starch into a multiphase polymeric material, using a melt compounding sequence, which can be irreversibly shaped by thermoforming for rethinked, sustainable packaging proved to ensure the materials needed and the specific properties required for such an application.

The properties of the selected quaternary compound and its thermoforming performance attest that due to the chemical compatibility between the main components, it was possible to destroy the hydrogen bonds established between the starch and PVOH macromolecules, overcome the inconveniences induced by the high content of amylopectin and the relative wide distribution of particle sizes of the mineral filler, and to establish new secondary bonds among the polar groups, mainly hydroxyl in nature, of the components from the selected quaternary compound. The resulted, comfortable, good miscibility between the compound components was also the consequence both of selecting the optimal embedding procedure and finding the appropriate range of compounding conditions. It has been found that low and medium speeds favour both the pre-gelling process as well as the melt homogenization. The content of branched polymers and fillers with relative variable size distribution affected the melt fluidity, its flow resistance and therefore the stationary flow, the required energy to flow and by default, the quality of the obtained thermoformed items. By adjusting the formulation, the selected compounding procedure and the pressing conditions, the unstable flow has been avoided, which was reflected by the smooth surface both of the extrudates and the pressed plates. As the plates obtained from the selected quaternary compound had reproducible mechanical properties, the chosen starch structuring solution covered the requirements of a thermoformable polymeric material, including the diminishing of the high degradability of the starch–PVOH compounds [9,41,42,43,48,59].

Due to the two fillers, it was possible to increase the glass transition from −6.4 °C, for the plasticized binary blends, to over 100 °C for the tertiary and quaternary compounds, (values settled by the DSC and DMA results), fulfilling, in this way, an essential condition for a polymeric material to be thermoformed. Additionally, due to the presence of the two fillers, the ratio between the elastic and the plastic deformation has been adjusted in such a way that thermoforming was possible. If the binary blends cannot be thermoformed because they do not deform elastically on the positive temperature range, the ternary and quaternary compounds can be processed into good items by this technique. For these tertiary and quaternary selected compounds, a good ratio between the elastic and viscous deformations was possible to be ensured so that, on the positive range of temperature these materials could be elastically deformed, without breaking.

The good miscibility between the components of the selected compounds is made clear by several strong proofs. Incontestable evidence of the good miscibility is the compact nature of the morphologies of the ternary compounds and of the quaternary selected ones by comparison with the granular morphology of the starch–PVOH binary blend. The SEM micrographs of this selected quaternary compound show very few or no defects as voids, cracks, fractures and the stress–strain curves are no longer dispersed. If the morphologies of quaternary and ternary compounds are compared, the role of wood flour in reducing the defects number is obvious, the ternary compositions having slightly more such deficiencies. If the starch-based binary blends have a clear biphasic morphology with starch granule residues represented by the branched amylopectin spread into the matrix formed by the two linear polymers, amylose and PVOH, in the case of the quaternary starch-based compounds, at the boundary between the phases appeared in an area with modified chemical composition, similar to those of the main matrix. Further undeniable proof of the good component’s miscibility is the thermal degradation behaviour of the selected compound in a mechanism with a single step and 2 small shoulders, in contrast to all the studied binary and ternary blends which degrade after a mechanism in two steps, the reproducibility of the mechanical properties, the stretching without breaking, the dimensional stability after stretching. The shape of the stress–strain curves allowed the identification of the possibilities to control with the help of the formulation the extensibility so that the new starch-based materials to be thermoformed in good conditions. The study of the melt rheological properties showed that the polymeric compounds based on filled starch are characterized by high melt resistance to flow and therefore intensive efforts are needed for homogenization of the melt thus avoiding the flow instability which strongly affects the quality of the obtained items.

The positive role of wood flour was distinguished in that it improved the melt flow, generated a strong decrease in the number of morphological defects and changed the mechanism of the thermal degradation from one with two-stage for tertiary blends to one with one stage, which means that it had an obvious compatibilizer role. The only explanation for the degree of miscibility reached is that through the used starch structuring solution, most likely, it was possible to place the fillers at the interface between various phases where they act as compatibilizers, having a main role in structuring the new materials. Otherwise, in the polymer compounds, practice it is accepted that the fillers play the role of a compatibilizer by lowering the interfacial tension, promoting the disintegration of the dispersed phase droplets, while avoiding the restoration of the droplets and stabilizing the system [45].The efficiency of fillers as compatibilizers depends on many factors [45].

The ratio between the elastic and the plastic deformation, the ability to absorb deformation energy which characterize the selected quaternary compound, allowed it to stretch without breaking and perform thermoforming in good condition, so that the obtained items had a good dimensional stability while maintaining its shape for more than 1 year. Near the linear polymers from the selected quaternary blend, which can be easily stretched, it is possible that the short ramifications from the amylopectin clusters are able to stretch as well, thus contributing to maintaining the shape of the obtained thermoformed object.

The manufacture of thermoformable packaging from the selected new starch-based material, achievable according to the presented solution, will bring compostable rethinked grades to the market [39,57] that will no longer accumulate into the environment, cover certain consumer values without endangering the environment [58], can be returned into usage as they keep their shape for at least 1 year after manufacture, allow pre-consumer and post-consumer wastes to be mechanically recycled according to the defects dilution principle [60,61] and can easily be composted [52,56,60,61,62,63,64,65]. The inorganic filler will be found in the composting products that will finally reach the ground after the compost feeds the plants.

Future will aim to obtain rethinked packaging for applications that require higher strength properties achievable, firstly, by including nanometric fillers [40] in a more elastic starch-based compound matrix and/or reactive compounding [9].

## 5. Conclusions

1. The presented structuring solution for starch conversion, through a melt compounding sequence, into a multiphase polymeric material which can be irreversibly shaped by thermoforming for rethinked, sustainable packaging has considered the physical modification of starch with PVOH, an inorganic target filler, CaCO_3_, a renewable compostable target filler, soft short wood fibre, and a good plasticizer compatible with the other polar components, such as glycerol.
2. The proposed structuring solution was studied through analysing the variation of the new compound’s properties with respect to the composition and the obtaining conditions, considering their values both in a solid (thermal, dynamic-mechanical, mechanical, dimensional stability, thermoformability, time behaviour of the selected quaternary blend) and melted state (fluidity, melt resistance to flow). The optimal compound was those with the composition which makes possible the thermoforming at 80–100 °C, assures a good ratio between the elastic and the plastic deformation and the resulting object does not break, degrade at compounding or at plates obtaining. It also possesses the mechanical properties required by the application and has the dimensional stability essential for the obtained product. The conditions in which the extrudates and the pressed plates had smooth surfaces did not present any other signs of flow instability and were considered as optimal for flowing in the melted state. The melt embedding variants and working conditions that led to reproducible mechanical properties and to the smallest number of morphological defects as voids were selected as optimal for obtaining the new material based on starch.
3. The properties, of the selected quaternary compound, and its thermoforming performance attest that, by using the proposed structuring solution, it was possible to overcome the impediments generated by the branched degree of the amylopectin from starch and the relatively wide range of the particles’ size distribution of the used fillers and to reach a comfortable miscibility as a result of the new settled affinities and, therefore, new secondary bonds among the used modification components.
4. The settling of new secondary bonds among the polar groups, mainly of hydroxyl nature, was possible following chemical compatibility between the used modification components and both the right choice of optimal embedding procedure for the fillers into the main polymeric matrix and of the range of melt compounding conditions.
5. The only explanation for the degree of miscibility reached in the case of the selected quaternary compound is that following the used starch structuring solution, the wood flour was layered at the interface between the phases where it acts as a compatibilizer with a central role in structuring of the new material. The status of wood flour as compatibilizer has been demonstrated by the improvment in the melt flow properties, the strong decrease in the number of morphological defects, changes in the thermal degradation mechanism from one with two stages for binary and tertiary blends, to one with one stage in the case of the selected quaternary compound, the reproducibility of the mechanical properties, stretching without breaking, and dimensional stability after stretching.
6. The thermoformed packaging made according to the presented structuring solution meets the conditions imposed by the imperatives of the present day, as they are compostable, cover certain consumer values without endangering the environment, can be returned into re-usage because they keep their shape and functional properties for at least 1 year after manufacture, and the pre-consumer and post-consumer wastes can be mechanically recycled according to the defects dilution principle in the manufacture of plates for thermoforming.

## Figures and Tables

**Table 1:** Extrusion procedures for improving the component miscibility.

Procedure (Screw Type and Extrusion Rate, rpm)			
I		II	
Stage		Stage	
Pre-gelling (1)	Homogenization (2)	Pre-gelling (1)	Homogenization (2)
Conveying screw	Compression screw	Conveying screw	Compression screw
150	20/40/70	5/20/50	40

**Table 2:** The transitions between −87 °C and 90 °C of the binary starch-based blends.

Plasticizer Content (%)/T Transition (°C)	Negative Range of Temperature (°C)						Positive Range of T (°C)			Melting Enthalpy (J/g)
	Transition 1			Transition 2			Transition 3			
	Onset	Midpoint	End Set	Onset	Midpoint	End Set	Onset	Midpoint	End Set	
**27%**	−80	−69.5	−60	−75	−57.9	−38	51	57	59	0.219
**35%**	−85	−78.7	−61	−74	−64.7	−50	51	55.1	58	0.342
**42%**	−87	−81.1	−74	−76	−69.2	−58	50	53.8	58	0.407

**Table 3:** The glass transitions of the plasticized starch-PVOH blends between 39 °C and 101 °C.

Plasticizer Content (%)/T Transition (°C)	Positive Range of T (°C)								
	Transition 1			Transition 2			Transition 3		
	Onset	Midpoint	End Set	Onset	Midpoint	End Set	Onset	Midpoint	End Set
**27%**	40	51.5	61	80.8	84.8	94	93	98.3	101
**35%**	40	47.2	57	76	81	85	90.7	94.9	97
**42%**	39	50	57	73	74	81	88	93.4	95

**Table 4:** Dependence of the thermal transitions and of the related enthalpies of the PVOH-starch compounds with variable CaCO_3_ content.

CaCO_3_ Content, %	T_evap_, °C	ΔH_evap_, J/g	T_crist_, °C	ΔH_crist_, J/g	T_melt._ °C	ΔH_melt._ J/g	T_g_, °C
**0**	122.3	170	120.3	75.6	180.8	104.4	−6.4
**2**	106.8	169.3	131.7	23.9	188.5	96.1	138.7
**5**	115.3	155.8	128.8	22.4	185/236.6	94.71	135.9
**9**	85.2	145.8	140.2	21.3	190.7	72.1	138.3
**23**	87.0	108.5	142.5	19.2	194.3	72.6	141.1
**50**	95.4	97.5	151.0	16.4	202.5	35.7	148.2
**67**	92.6	91.3	153.5	15.3	202.7	16.0	169.4
**PVOH**	73.4	154.9	102.5	21.8	-	-	68
**Starch**	88.4	415.0	-	-	-	-	70
**CaCO_3_**			-		186.6	4.38	-

**Table 5:** Dependence of the thermo–oxidative degradability of the starch–PVOH blends on the CaCO_3_ quantity.

CaCO_3_, [%]	T_d3%_, °C	T_d50%_, °C	T_max1_, °C	T_max2_, °C	Weight Loss, % (20 ÷ 500 °C)
**0**	85.5	270.2	214.5	325.4	94.2
**2**	83.2	272.3	205.6	314.8	92.7
**5**	101.2	283.6	209.7	325.0	91.8
**9**	96.4	275.9	201.2	312.4	89.2
**23**	104.2	305.9	212.0	321.1	78.7
**50**	116.4	310.9	209.6	302.3	55.8
**67**	131.8	-	203.6	308.8	39.3
**PVOH**	236.0	318.1	315.2	450.2	94.1
**Starch**	97.53	320.1	-	321.0	90.4
**CaCO_3_**	-	-	-	407.6	0.96

**Table 6:** Thermal behaviour of compounds with and without wood flour and the same CaCO_3_ amount.

Compound Filler’s Type	Melting			Crystallization		
	Range, °C	Maximum, °C	ΔHmelt. J/g	Range, °C	Maximum, °C	ΔHmelt. J/g
**CaCO_3_**	170–220	200	55	140–180	149	19
**CaCO_3_ + WF**	120–200	181.6	7.03	90–200	145.8	15

**Table 7:** The flow melt properties of the quaternary selected blend with CaCO_3_ and 30% wood flour.

Temperature, °C	Property	Weight, kg (Ternary Blend with 37% CaCO_3_ and 30% WF)			
		2.16	3.8	5	10
**145**	Fluidity, g/10′	0.440	1.028	2.056	10.98
	Dynamic viscosity (η), Pa∙s	22,809.40	16,155.90	13,765.20	6344.00
	Shear rate	0.86	2.12	3.28	14.24
**155**	Fluidity, g/10′	3.056	6.164	7.924	33.32
	Dynamic viscosity (η), Pa∙s	9836.30	5604.30	4750.30	2214.20
	Shear rate	1.98	6.12	9.51	40.79
**165**	Fluidity, g/10′	4.604	6.808	9.972	36.93
	Dynamic viscosity (η), Pa∙s	10,246.90	5234.90	3294.70	3128.30
	Shear rate	1.90	6.56	13.71	28.87
**175**	fluidity, g/10′	5.812	9.948	9.84	37.85
	Dynamic viscosity (η), Pa∙s	7545.10	4947.30	3463.30	1638
	Shear rate	2.59	6.94	13.04	55.14

## Data Availability

The data presented in this study are available on request from the corresponding author.
